# Physical Activity Pattern of Adults With Metabolic Syndrome Risk Factors: Time-Series Cluster Analysis

**DOI:** 10.2196/50663

**Published:** 2023-12-01

**Authors:** Junhyoung Kim, Jin-Young Choi, Hana Kim, Taeksang Lee, Jaeyoung Ha, Sangyi Lee, Jungmi Park, Gyeong-Suk Jeon, Sung-il Cho

**Affiliations:** 1 Department of Public Health Science, Graduate School of Public Health, Seoul National University, Seoul, Republic of Korea; 2 Department of Nursing, Mokpo National University, Muan, Republic of Korea; 3 Institute of Health and Environment, Seoul National University, Seoul, Republic of Korea

**Keywords:** wrist-worn wearable, wearable data, physical activity pattern, TADPole clustering, TADPole, cluster, clustering, wearable, wearables, wrist-worn, physical activity, pattern, patterns, data analysis, data analytics, regression, risk, risks, time series, Time-Series Anytime Density Peak

## Abstract

**Background:**

Physical activity plays a crucial role in maintaining a healthy lifestyle, and wrist-worn wearables, such as smartwatches and smart bands, have become popular tools for measuring activity levels in daily life. However, studies on physical activity using wearable devices have limitations; for example, these studies often rely on a single device model or use improper clustering methods to analyze the wearable data that are extracted from wearable devices.

**Objective:**

This study aimed to identify methods suitable for analyzing wearable data and determining daily physical activity patterns. This study also explored the association between these physical activity patterns and health risk factors.

**Methods:**

People aged >30 years who had metabolic syndrome risk factors and were using their own wrist-worn devices were included in this study. We collected personal health data through a web-based survey and measured physical activity levels using wrist-worn wearables over the course of 1 week. The Time-Series Anytime Density Peak (TADPole) clustering method, which is a novel time-series method proposed recently, was used to identify the physical activity patterns of study participants. Additionally, we defined physical activity pattern groups based on the similarity of physical activity patterns between weekdays and weekends. We used the *χ*^2^ or Fisher exact test for categorical variables and the 2-tailed *t* test for numerical variables to find significant differences between physical activity pattern groups. Logistic regression models were used to analyze the relationship between activity patterns and health risk factors.

**Results:**

A total of 47 participants were included in the analysis, generating a total of 329 person-days of data. We identified 2 different types of physical activity patterns (early bird pattern and night owl pattern) for weekdays and weekends. The physical activity levels of early birds were less than that of night owls on both weekdays and weekends. Additionally, participants were categorized into stable and shifting groups based on the similarity of physical activity patterns between weekdays and weekends. The physical activity pattern groups showed significant differences depending on age (*P*=.004) and daily energy expenditure (*P*<.001 for weekdays; *P*=.003 for weekends). Logistic regression analysis revealed a significant association between older age (≥40 y) and shifting physical activity patterns (odds ratio 8.68, 95% CI 1.95-48.85; *P*=.007).

**Conclusions:**

This study overcomes the limitations of previous studies by using various models of wrist-worn wearables and a novel time-series clustering method. Our findings suggested that age significantly influenced physical activity patterns. It also suggests a potential role of the TADPole clustering method in the analysis of large and multidimensional data, such as wearable data.

## Introduction

Physical activity has been linked to numerous health benefits. A cross-sectional study conducted in Japan reported that inactive individuals with <23 metabolic equivalent (MET) hours per week had more than double the risk of metabolic syndrome compared to active individuals (≥23 MET h/wk) [1]. Another study used X-means clustering to identify intensity and temporal activity patterns and demonstrated that inactive individuals had a 3-fold higher risk of cardiovascular disease compared to active individuals [2]. A systematic review also suggested that increased physical activity correlates with improved health status [3]. Additionally, a meta-analysis by Pearce et al [4] found that adults who achieved the recommended physical activity level (4.4 marginal MET h/wk) had a 25% lower risk of depression compared to inactive adults.

Wrist-worn wearables, such as smartwatches and smart bands equipped with computers and sensors, have become popular tools for measuring physical activity [5,6]. Most individuals opt to wear wrist-worn wearables for several reasons, including affordability, functionality, and stylish design [7]. This has enabled the measurement of physical activity in daily life rather than being limited to the laboratory setting. There have also been notable improvements in the accuracy of measurements obtained from wrist-worn wearables [8,9]. As a result, an increasing number of studies are focusing on measuring and analyzing physical activity using wrist-worn devices [7].

Several studies are currently exploring different aspects of physical activity using wrist-worn devices. These include investigations of the accuracy of these devices [8,9], the relationship between physical activity and personal characteristics [2,10], the impact of interventions using wrist-worn wearables [11,12], and behavior prediction [13-15]. Some studies have also sought to identify physical activity patterns using data collected from wrist-worn wearables [2,16-20]. However, it is important to note that these studies have certain limitations.

The diversity of wearable device models poses a challenge for observational studies using wearables within a population. Most previous studies either provided the participants with a specific device model or restricted participation to individuals using a particular model [21-23]. However, wearable device models are continuously evolving to cater to individual preferences. Furthermore, each model has its own app, which extracts data in a specific format. Consequently, there is a need for flexible methods that can effectively analyze essential information derived from diverse forms of wearable data.

Grouping methods, such as principal component analysis [16-18] and *k*-means clustering [2,19,20], have commonly been used to identify similarities among participants and summarize activity patterns within groups. Time-series analysis methods can also be used to classify daily activity patterns. However, previous studies using popular clustering methods have shown sensitivity to minor variations in data formats, resulting in inconsistent outcomes.

The *k*-means clustering method is a kind of partitional clustering method [24]. This clustering method is easy to implement and successfully distinguishes clusters using data from all participants, with low computational cost [20,24]. Therefore, it can be applied to large and multidimensional data. However, the number of clusters (parameter *k*) had to be predefined, because the parameter *k* is not commonly known; therefore, iterative analysis is required to get the optimal number of clusters. The X-means clustering mentioned above is also a type of *k*-means clustering and is a clustering method that automatically finds the number of clusters by taking the disadvantage of *k*-means into consideration [2]. In addition, the *k*-means clustering method provides unstable results due to its random selection of the initial centroid [24].

The hierarchical clustering method and Density-Based Spatial Clustering of Applications With Noise (DBSCAN) are also popular time-series clustering methods [19,20]. The hierarchical clustering method is a method of classifying clusters based on the hierarchical structure of data and basically considers 1 time series as 1 cluster [20,24]. The hierarchical clustering method has the advantage of visualizing the hierarchical structure of data, because it shows the hierarchical structure as a tree (ie, a dendrogram). However, its computational cost is high, and a significant number of data points must be excluded from the analysis to obtain the desired number of clusters, raising uncertainties about the accuracy of the resulting clusters [24]. DBSCAN is a density-based clustering method that calculates the density of data based on the Euclidean distance calculation method and excludes data considered as noise from clustering. However, as shown in a study by Dobbins and Rawassizadeh [20], DBSCAN has the highest computational cost, and the Euclidean method applied to DBSCAN is not suitable for multidimensional data. Thus, the hierarchical clustering method and DBSCAN are not feasible for large or multidimensional data.

A more flexible time-series clustering method called Time-Series Anytime Density Peak (TADPole) clustering has recently been proposed [24,25]. This is an algorithm that can perform fast clustering by reducing the distance calculation process in the Density Peak clustering method. This method uses dynamic time warping to calculate distances between series. Unlike the Euclidean method, which matches data at the same points across a series, this method identifies optimal warping paths between series to identify better point-to-point matches except for the first and last points. The prototype for TADPole clustering is partition around medoid clustering, which creates clusters by minimizing the sum of distances calculated based on an arbitrary series (ie, medoid) [24]. TADPole clustering classifies series as neighbors if the distance between them is below a certain cutoff value [24,25]. Theoretical and technical details are readily accessible in the previous literature [24,25] and thus are not repeated here.

The TADPole clustering algorithm can cluster multidimensional data measured by wearable devices as well as large data [25]. In this study, we chose the TADPole clustering method to test whether it can be effectively applied to wearable data, which was not addressed in its published paper [25]. To the best of our knowledge, this novel approach has not yet been applied to the study of health indicators measured using wearable devices. Therefore, this study assessed the feasibility of using a time-series clustering method to analyze wearable data for daily physical activity patterns and explored the association between these patterns and health risk factors.

## Methods

### Study Participants

This study examined physical activity patterns among at-risk individuals using wrist-worn wearables. Step counts, distances, and energy expenditure (EE) were measured over 1 week in a real-life setting between November 22, 2021, and December 2, 2021. Participants aged >30 years who had risk factors based on metabolic syndrome diagnostic criteria and were currently using wrist-worn devices (eg, smartwatches and smart bands) were included. The risk factors included blood pressure ≥130/85 mm Hg, fasting blood sugar ≥100 mg/dL, triglyceride levels ≥150 mg/dL, high-density lipoprotein level <40 or <50 mg/dL (for male and female individuals, respectively), and waist circumference ≥90 or ≥85 cm (for male and female individuals, respectively). The number of study participants was selected based on the analysis results using G*Power (Heinrich Heine Universität Düsseldorf) and previous similar research cases. First, we used G*Power to perform an ANOVA because the data of the study participants would be measured repeatedly. The effect size (Cohen *f*) was set to be 0.25, and the significance level (α) and power (1 – β) were assumed to be 5% and 95%, respectively. As a result, a total sample size of 36 was calculated. Next, we considered the previous work by Huh et al [26], which applied wearable technology to patients with metabolic syndrome and recruited a total of 53 people. However, 33 patients dropped out during the 12-week study period due to the withdrawal of consent, device malfunction, and the loss of follow-up. We finally decided to recruit 60 study participants to achieve a sufficient effect. We used the Seoul National University mailing system to recruit research participants. Starting on November 3, 2021, we sent 2 emails to all members of the university; this lasted until November 19, 2021, when the recruitment was completed. The purpose of the study, eligibility criteria for study participants, and research procedures were provided via email. A detailed explanation of the study was provided in a web-based meeting after all written informed consent was obtained. Before physical activity measurements, all participants completed a web-based questionnaire through Google Forms, which collected personal health data and details about their physical activity (including type, intensity, and duration). The participants wore their own wrist-worn wearables for ≥10 hours per day with the physical activity measurement function activated for 1 week.

Following the 1-week period of physical activity measurements, the participants were asked to complete another web-based questionnaire to assess user experience, including cognition, context, applicability, and behavioral changes. Of the 60 participants initially included in the study, 13 were excluded due to missing or limited baseline data (n=3) or the unavailability of physical activity data from the database (n=10). Consequently, the analysis involved 47 participants who met the inclusion criteria and had data available for analysis.

### Data Collection

During the 7-day measurement period, the participants activated the measurement function on their wrist-worn wearables, generating a total of 329 person-days of data. The study included wrist-worn wearables from the Apple Watch, Samsung Galaxy Watch, and Xiaomi Mi Band series (Multimedia Appendix 1). Data were collected continuously throughout the measurement period, with each device automatically storing individual data and synchronizing it with the participants’ cell phones.

Upon the completion of the measurement period, each participant exported their individual data through the official data export system and submitted the data via email. We provided the participants with detailed instructions specific to the manufacturer, version, and brand of their wrist-worn wearables. We then decompressed the data files and preprocessed the data to extract the selected variables, including step count, distance, EE, and duration with the start and end points. These variables were then merged into a unified data format for analysis.

### Time-Series Clustering

The time-series clustering method was applied in 3 steps to identify clusters representing the physical activity patterns of the study participants. First, due to variations in data recording formats among the different wrist-worn device brands, the data were edited to ensure consistency. Data from different brands were standardized into the same format. For instance, the Samsung Galaxy Watch series record EE as “kcal per minute” during wearing, whereas the Apple Watch and Xiaomi Mi Band series record EE as “cal” and “kcal,” respectively, for each distinct activity. We converted these data into 10-minute “kcal” EE values. For activities performed for >10 minutes while wearing the Apple Watch or Xiaomi Mi Band series, the activity duration was divided into 10-minute intervals, assuming that a consistent amount of energy was expended during each interval.

**Figure 1.**
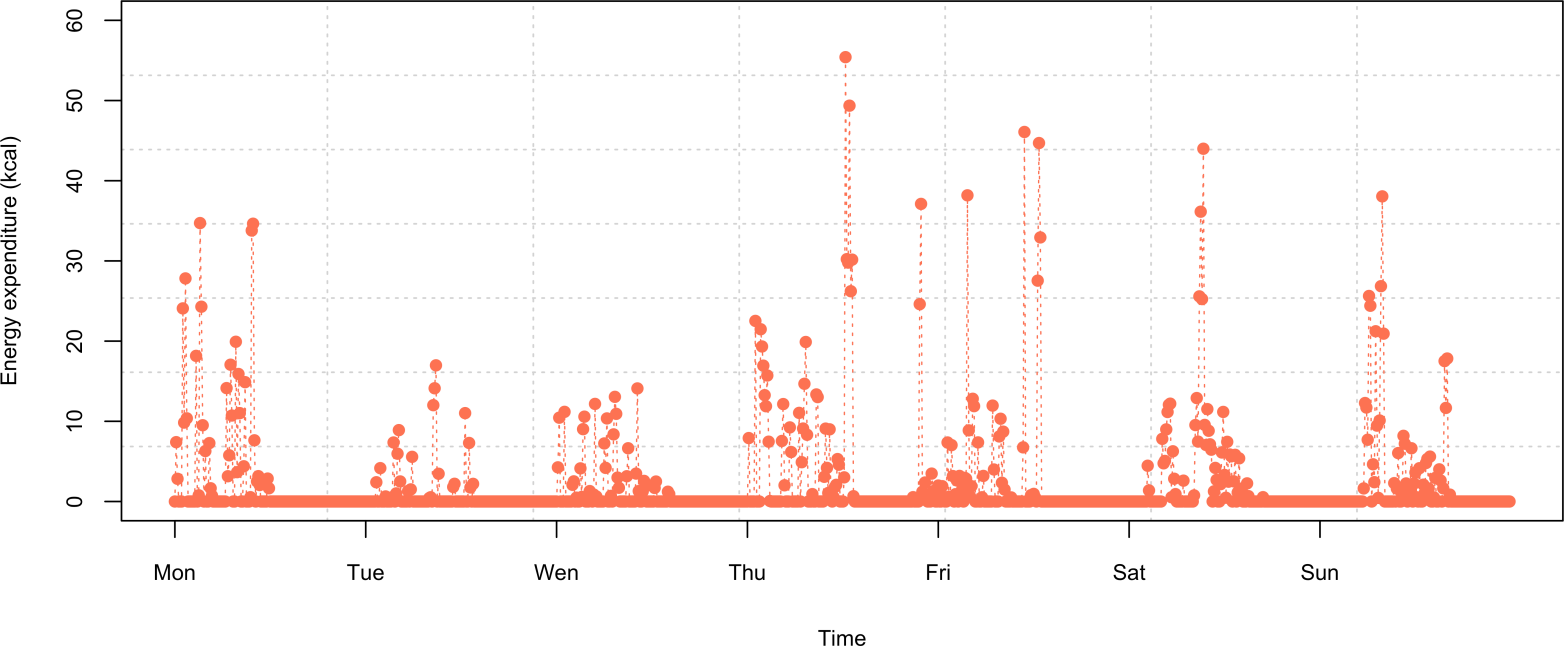
Physical activity measurement for 1 of the participants (7 person-days).

Second, the data were divided into weekdays (Monday to Friday; 235 person-days) and weekends (Saturday and Sunday; 94 person-days), with the clustering method applied separately to each group. We calculated the average EE for weekdays and weekends based on the daily 10-minute EE values. It was assumed that the EE was 0 when the participant was not wearing the device. Figure 1 illustrates an example of physical activity measurements for 7 person-days, with each data point representing the EE in kcal per 10 minutes over the course of 1 week.

Third, time-series clustering was conducted through TADPole clustering, a recently developed technique that allows faster clustering by implementing a cutoff value to determine clusters [24,25]. To determine the optimal clustering model, we analyzed the expected number of clusters (parameter *k*) and the cutoff value. For the third step, we used the *dtwclust* package in RStudio [27]. This package offers a range of functions for conducting time-series clustering, including the TADPole clustering method. We had to specify certain parameters, including the cluster type, the number of clusters, the cutoff value, and the window size. Since we chose TADPole as the cluster type, we did not need to specify the distance parameter. As for the cutoff value and window size, we adjusted them based on the volume of data. Given that our study used 144 data points, we selected values that fell below this threshold. As the optimal number of clusters and cutoff values were unknown, cluster evaluation was performed using the silhouette index, which is a popular cluster validity index. Based on the cluster evaluation, the model with the highest silhouette index was selected as the optimal clustering model.

### Statistical Analysis

The demographic characteristics of the participants, including sex, age, work type (sitting, standing, etc), daily EE (weekdays and weekends), physical activity changes after using wrist-worn wearables, weekly physical activity patterns, and number of risk factors (1 or >1), were recorded. For categorical variables, the number and proportion for each category were presented, along with the *P* value calculated using the *χ*^2^ or Fisher exact test for variables with counts <5. For numerical variables, mean and SD with *P *values were calculated using the 2-tailed *t* test.

The association between weekly physical activity patterns and participant characteristics was analyzed using a logistic regression model. The regression model was evaluated in terms of pseudo-*R*^2^, accuracy, Hosmer-Lemeshow goodness of fit, and the receiver operating characteristic curve. The results of the logistic regression were presented as odds ratios with 95% CIs and the corresponding *P* values. Statistical significance was taken as *P*≤.05. The statistical analyses were performed using RStudio (version 2022.07.2+576; Posit) [28].

### Ethical Considerations

The study was approved by the Institutional Review Board of Mokpo National University (approval MNUIRB-210625-SB-014-01). All participants provided informed consent before study participation. The submitted data were anonymized before analysis. Participants who provided data and finished the web-based survey received a compensation of ₩100,000 (US $77.65).

## Results

### General Participant Characteristics

Among the 47 participants, 23 (49%) were male and 24 (51%) were female (Table 1). In terms of age, 30 (64%) participants were aged <40 years, whereas 17 (36%) were aged ≥40 years. The majority (n=42, 89%) of the participants had a sedentary job. The average EE during weekdays was 223 (SD 175) kcal, whereas that on weekends was 191 (SD 164) kcal. After using wrist-worn wearables, 29 (62%) participants reported a decrease or no change in physical activity, whereas 18 (38%) participants reported an increase. In terms of health risk factors, 25 (53%) participants had only 1 risk factor, whereas 22 (47%) had >1 risk factors.

**Table 1.** General participant characteristics.

Variable and level		Value (N=47)
**Sex, n (%)**		
	Male	23 (49)
	Female	24 (51)
**Age group (y), n (%)**		
	<40	30 (64)
	≥40	17 (36)
**Work type, n (%)**		
	Sitting	42 (89)
	Other	5 (11)
**Daily EE**^**a**^ **(kcal), mean (SD)**		
	Weekdays	223 (175)
	Weekends	191 (164)
**Change in PA** ^**b**^ **, n (%)**		
	No change or decrease	29 (62)
	Increase	18 (38)
**PA** **pattern group, n (%)**		
	Stable	35 (75)
	Shifting	12 (25)
**Number of health risk factors, n (%)**		
	1	25 (53)
	>1	22 (47)

a EE: energy expenditure.

b PA: physical activity.

### Physical Activity Patterns

**Figure 2.**
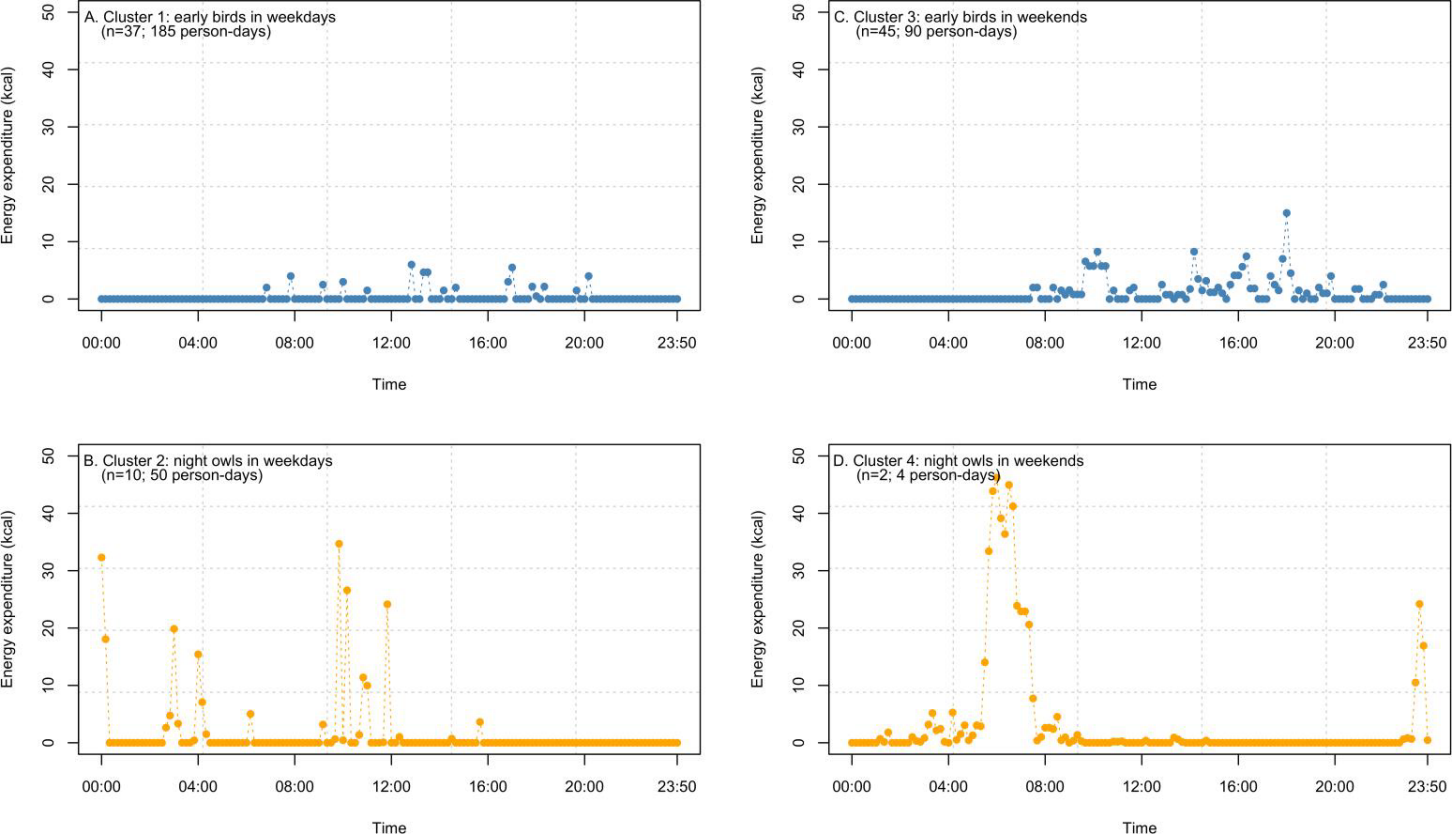
Physical activity clusters on weekdays and weekends: (A) cluster 1: “early birds” on weekdays, (B) cluster 2: “night owls” on weekdays, (C) cluster 3: “early birds” on weekends, and (D) cluster 4: “night owls” on weekends.

The time-series cluster analysis resulted in the highest silhouette index when there were 2 clusters (*k*=2) for both weekdays and weekends. Therefore, 2 clusters each were distinguished for weekdays and weekends (Figure 2). The left and right columns of Figure 2 represent the weekday and weekend clusters, respectively. Each cluster included data from at least 2 participants, and 2 distinct cluster types were distinguished with different starting times for physical activity. The “early bird” type (represented by blue dots in Figure 2) initiated physical activity after 6 AM, whereas the “night owl” type (represented by orange dots in Figure 2) began physical activity before 6 AM.

Among the 37 early birds on weekdays, 57% (n=21) were female and 76% (n=28) were in their 30s, with female participants in their 30s accounting for the highest proportion (n=18, 48%). Among the 10 night owls on weekdays, 70% (n=7) were male and 80% (n=8) were in their aged ≥40 years, and 40% (n=4) were male individuals aged ≥40 years. Weekend physical activity patterns were mostly from early birds (n=45, 96%) regardless of sex or age. Out of the 45 early birds on weekends, 51% (n=23) were female and 64% (n=29) were in their 30s, and 40% (n=18) were female individuals aged <40 years. There was 1 participant per sex and age group who was a night owl on weekends, and there was no one who was a night owl on both weekdays and weekends.

Figure 2A shows the physical activity patterns of the 37 (79%) out of 47 participants belonging to cluster 1 (early birds) on weekdays. Physical activity occurred between 6 AM and 8 PM, with most activities being <10 kcal (mean 2.98 kcal). Meanwhile, cluster 2 included 10 (21%) “night owls” on weekdays (Figure 2B). Physical activity for cluster 2 typically started at midnight and ended before 4 PM. Cluster 2 also exhibited greater EE, with an average of 9.51 kcal.

During the weekends, the majority (45/47, 96%) of participants were early birds (cluster 3; Figure 2C). Cluster 3 had an average EE of 2.94 kcal. Cluster 4 (night owls) included only 2 (4%) participants on weekends (Figure 2D) and demonstrated a higher EE (average 8.60 kcal).

Although the average EE was similar between early birds and night owls, their physical activity patterns differed on weekdays and weekends. Regardless of the cluster type, physical activity tended to be shorter in duration on weekdays (Figure 2A and B), becoming longer and more continuous on weekends. Cluster 3, representing early birds on weekends, exhibited up to 10 consecutive physical activity periods, which is equivalent to 100 minutes (4-8 AM; Figure 2C). Cluster 4, representing the night owls on weekends, had the highest total EE of 507.59 kcal and up to 16 consecutive physical activity periods, which is equivalent to 160 minutes (4-8 AM; Figure 2D).

Based on the analysis of physical activity patterns, 2 groups were identified: the stable group and the shifting group. The stable group included individuals who maintained the same physical activity pattern on weekdays and weekends, regardless of whether they were classified as early birds or night owls. Among the 47 participants, 35 (74%) belonged to the stable group, exhibiting early bird physical activity patterns consistently throughout the week. There were no participants belonging to both clusters 2 and 4. On the other hand, the shifting group included individuals whose physical activity patterns differed between weekdays and weekends. There were 12 (26%) participants in the shifting group; 10 (21%) participants displayed an early bird pattern during weekdays (cluster 1) but changed to the night owl pattern on weekends (cluster 4). The remaining 2 (4%) participants exhibited the opposite pattern, that is, a night owl pattern during weekdays (cluster 4) and an early bird pattern on weekends (cluster 1).

Demographic descriptive statistics for the physical activity pattern groups, including the results of the *χ*^2^ test for categorical variables and the *t* test for continuous variables, are presented in Table 2. There were no significant differences between the physical activity pattern groups except in age (*P*=.001) and EE (*P*<.001 for weekdays; *P*=.003 for weekends).

**Table 2.** Weekly physical activity (PA) group characteristics.

Variable and level		PA pattern group		*P* value
		Stable (n=35)	Shifting (n=12)	
**Sex, n (%)**				.19^a^
	Male	15 (43)	8 (67)	
	Female	20 (57)	4 (33)	
**Age group (y), n (%)**				.004^a^
	<40	27 (77)	3 (25)	
**Work type, n (%)**				>.99^a^
	Sitting	31 (89)	11 (92)	
	Other	4 (11)	1 (8)	
**Daily EE**^**b**^ **(kcal), mean (SD)**				
	Weekdays	169 (130)	383 (194)	<.001^c^
	Weekends	145 (100)	327 (230)	.003^c^
**PA changes, n (%)**				.74^a^
	No change or decrease	21 (60)	8 (67)	
	Increase	14 (40)	4 (33)	
**Number of health risk factors, n (%)**				.18^a^
	1	21 (60)	4 (33)	
	>1	14 (40)	8 (67)	

a Fisher exact test.

b EE: energy expenditure.

c *t* test.

### Association Between Physical Activity Patterns and Health Risk Factors

A logistic regression model was used to examine the associations of sex, age, and the number of health risk factors with weekly physical activity patterns (Table 3). Logistic regression model accuracy and diagnostic results are presented in Multimedia Appendix 1. Sex (*P*=.45) and the number of health risk factors (*P*=.33) were not significantly associated with the physical activity pattern. In contrast, age showed a statistically significant association with physical activity patterns; the higher age group had higher odds of differences between weekday and weekend physical activity patterns (odds ratio 8.68, 95% CI 1.95-48.85; *P*=.007).

**Table 3.** Associations between physical activity patterns and health risk factors.

Variable and level (reference)	OR^a^ (95% CI)		*P* value
Sex: female (vs male)	0.69 (0.13-3.64)		.45
Age group: ≥40 y (vs <40 y)	8.68 (1.95-48.85)		.007
Number of health risk factors: >1 (vs 1)	2.21 (0.45-11.92)		.33

a OR: odds ratio.

To account for the possibility of reverse causality, we conducted another logistic regression analysis with the number of health risk factors as the outcome variable. Despite this adjustment, there were no significant associations between physical activity patterns and health risk factors (*P*>.99; Multimedia Appendix 1).

## Discussion

### Principal Findings

In this study, we assessed the effectiveness of the TADPole clustering method for identifying physical activity patterns from wearable data. We also explored the association between these patterns and health risk factors. We found that physical activity patterns on weekdays and weekends were categorized as either daytime (early bird) or nighttime (night owl) patterns. Furthermore, 2 groups were distinguished: 1 with consistent physical activity patterns on weekdays and weekends (stable group) and the other with different patterns between weekdays and weekends (shifting group). Age significantly influenced physical activity patterns.

### Comparison to Prior Works

We found that physical activity patterns on weekdays and weekends differed as age increased. Our findings shed light on previously unaddressed or overlooked associations between physical activity patterns and health risk factors. Many previous studies did not report the association between age and physical activity patterns [17,18], and this association was reported only in a few studies [29-32]. Some of these studies reported results consistent with our findings. A study by Caspersen et al [29], which analyzed physical activity patterns according to sex and age, found that not only inactivity but also vigorous activity increased with age. In their study, those aged 18-29 years showed a pattern with the lowest vigorous activity and the highest sustained physical activity, whereas those aged ≥75 years showed a pattern with the highest vigorous activity and the lowest sustained physical activity, indicating differences in physical activity patterns by age. Another study by Rossen et al [32], which analyzed the physical activity patterns of individuals with diabetes for 2 years, found that the younger the age, the more physical activity increased 2 years later, showing that physical activity patterns can vary depending on age.

The physical activity patterns identified in our study were similar to chronotypes, which categorize individuals into morning type (M-type), evening type (E-type), and intermediate type (N-type) based on their preferred timings of activities and sleep [33]. M-type individuals are typically early birds, whereas E-type individuals are night owls who prefer late activity and sleep schedules. N-type individuals do not fall strictly into either category, and most adults belong to this type [33]. Previous studies have suggested that E-type individuals have lower physical activity levels and a higher risk of metabolic syndrome [34,35]. However, this study found that the individuals with night owl tendencies, that is, E-type individuals, exhibited higher physical activity levels compared to early birds, that is, M-type individuals. It is important to note that most participants in our study reported engaging in sedentary work, indicating that the increased physical activity levels among night owls were likely due to leisure activities rather than occupational tasks. This suggests that physical activity patterns are not determined solely by chronotype and that other factors, such as health awareness, can have a significant impact. It is worth noting that an individual’s chronotype and activity times may vary based on age and occupation, potentially leading to health issues if not addressed [36]. Therefore, it is crucial to make efforts to achieve the recommended level of physical activity regardless of the specific activity pattern or chronotype.

None of the participants in our study met the criteria for “weekend warriors,” which refers to individuals engaging in 1 or 2 sessions of physical activity, particularly on weekends, per week, consuming at least 1000 kcal [37]. Although the combined EE for cluster 2 (weekday night owls) and cluster 4 (weekend night owls) was the closest to that of weekend warriors at 735 kcal, no participants were included in both clusters. In a study conducted by Jang et al [38] in South Korea, only 2.1% of the participants were classified as weekend warriors, but there was no significant difference in metabolic risk between weekend warriors and the regularly active group. The weekend warrior physical activity pattern, which is popular in the United Kingdom, the United States, and Latin America [39], is associated with several health benefits, including a lower risk of obesity [38] and all-cause mortality [37]. A study of Chinese adults found that the weekend warrior physical activity pattern was associated with a lower risk of metabolic syndrome, hypertension, and diabetes in both male and female individuals [40]. Promoting physical activity guidelines may increase the number of weekend warriors in South Korea, where sedentary jobs are common (Table 1).

The TADPole clustering method addresses the limitations of previously popular time-series clustering techniques such as the *k*-means and hierarchical clustering methods in data analysis. As we mentioned in the introduction, the *k*-means clustering method often produced unstable results with clusters changing each time, whereas the TADPole clustering method consistently provided reliable clustering outcomes. However, similar to *k*-means, we iteratively performed the analysis to obtain the optimal clustering results. The TADPole clustering method stands out by providing more reliable results compared to hierarchical clustering and DBSCAN, as it uses all available data without any loss or exclusion. Additionally, our study demonstrated the feasibility of the TADPole clustering method, showing its suitability for handling large and multidimensional data such as wearable data.

### Strengths

A key advantage of our study was that we used multiple wrist-worn device models. Although wearables provide data in different formats depending on the model, we standardized the data into a single format to successfully conduct statistical analyses. Furthermore, the TADPole clustering method allowed us to overcome the limitations of hierarchical and *k*-means clustering, which are commonly used time-series clustering methods, resulting in robust and reliable findings.

### Limitations

This study had several limitations. It only included 47 participants, which may not have been sufficient to generate meaningful results. The measurement period for physical activity was only 7 days, which may not have been representative of the daily physical activity. Additionally, we assumed that the EE for physical activities exceeding 10 minutes was consistent across 10-minute intervals. Although these assumptions may not perfectly reflect reality, they were considered reasonable given that the participants were going about their normal daily routines.

### Conclusions

This study successfully performed time-series clustering using various wrist-worn device models and found TADPole clustering to be a suitable tool for analyzing the data. Physical activity patterns on weekdays and weekends could be categorized into “early birds” and “night owls,” and these patterns were significantly influenced by age. To address the limitations of our study, additional studies with larger sample sizes are required.

## Supplementary material

10.2196/50663 Multimedia Appendix 1 Included wearable models and the results of the regression model.

## Abbreviations

DBSCAN: Density-Based Spatial Clustering of Applications With Noise
E-type: evening type
EE: energy expenditure
M-type: morning type
MET: metabolic equivalent
N-type: intermediate type
TADPole: Time-Series Anytime Density Peak

## References

[1] Kim J, Tanabe K, Yokoyama N, Zempo H, Kuno S (2011). Association between physical activity and metabolic syndrome in middle-aged Japanese: a cross-sectional study. BMC Public Health.

[2] Niemelä M, Kangas M, Farrahi V (2019). Intensity and temporal patterns of physical activity and cardiovascular disease risk in midlife. Prev Med.

[3] Warburton DER, Bredin SSD (2017). Health benefits of physical activity: a systematic review of current systematic reviews. Curr Opin Cardiol.

[4] Pearce M, Garcia L, Abbas A (2022). Association between physical activity and risk of depression: a systematic review and meta-analysis. JAMA Psychiatry.

[5] Al-Eidan RM, Al-Khalifa H, Al-Salman AM (2018). A review of wrist-worn wearable: sensors, models, and challenges. J Sens.

[6] Nelson BW, Low CA, Jacobson N, Areán P, Torous J, Allen NB (2020). Guidelines for wrist-worn consumer wearable assessment of heart rate in biobehavioral research. NPJ Digit Med.

[7] Huhn S, Axt M, Gunga HC (2022). The impact of wearable technologies in health research: scoping review. JMIR Mhealth Uhealth.

[8] O’Driscoll R, Turicchi J, Hopkins M, Horgan GW, Finlayson G, Stubbs JR (2020). Improving energy expenditure estimates from wearable devices: a machine learning approach. J Sports Sci.

[9] Davoudi A, Wanigatunga AA, Kheirkhahan M (2019). Accuracy of Samsung Gear S smartwatch for activity recognition: validation study. JMIR Mhealth Uhealth.

[10] Biddle SJH, Bengoechea EG, Pedisic Z, Bennie J, Vergeer I, Wiesner G (2017). Screen time, other sedentary behaviours, and obesity risk in adults: a review of reviews. Curr Obes Rep.

[11] Pope ZC, Barr-Anderson DJ, Lewis BA, Pereira MA, Gao Z (2019). Use of wearable technology and social media to improve physical activity and dietary behaviors among college students: a 12-week randomized pilot study. Int J Environ Res Public Health.

[12] Gonzalez-Plaza E, Bellart J, Arranz Á, Luján-Barroso L, Mirasol EC, Seguranyes G (2022). Effectiveness of a step counter smartband and midwife counseling intervention on gestational weight gain and physical activity in pregnant women with obesity (Pas and Pes study): randomized controlled trial. JMIR Mhealth Uhealth.

[13] Fuller D, Anaraki JR, Simango B (2021). Predicting lying, sitting, walking and running using Apple Watch and Fitbit data. BMJ Open Sport Exerc Med.

[14] O’Driscoll R, Turicchi J, Hopkins M (2021). Comparison of the validity and generalizability of machine learning algorithms for the prediction of energy expenditure: validation study. JMIR Mhealth Uhealth.

[15] Sucerquia A, López JD, Vargas-Bonilla JF (2018). Real-life/real-time elderly fall detection with a triaxial accelerometer. Sensors (Basel).

[16] Mesquita R, Spina G, Pitta F (2017). Physical activity patterns and clusters in 1001 patients with COPD. Chron Respir Dis.

[17] Stenholm S, Pulakka A, Leskinen T (2021). Daily physical activity patterns and their association with health-related physical fitness among aging workers-the Finnish Retirement and Aging Study. J Gerontol A Biol Sci Med Sci.

[18] Albalak G, Stijntjes M, Wijsman CA (2022). Timing of objectively-collected physical activity in relation to body weight and metabolic health in sedentary older people: a cross-sectional and prospective analysis. Int J Obes (Lond).

[19] Jones PJ, Catt M, Davies MJ (2021). Feature selection for unsupervised machine learning of accelerometer data physical activity clusters - a systematic review. Gait Posture.

[20] Dobbins C, Rawassizadeh R (2018). Towards clustering of mobile and smartwatch accelerometer data for physical activity recognition. Informatics.

[21] Laranjo L, Ding D, Heleno B (2021). Do smartphone applications and activity trackers increase physical activity in adults? systematic review, meta-analysis and metaregression. Br J Sports Med.

[22] Kim Y, Wijndaele K, Sharp SJ (2019). Specific physical activities, sedentary behaviours and sleep as long-term predictors of accelerometer-measured physical activity in 91,648 adults: a prospective cohort study. Int J Behav Nutr Phys Act.

[23] Radin JM, Wineinger NE, Topol EJ, Steinhubl SR (2020). Harnessing wearable device data to improve state-level real-time surveillance of influenza-like illness in the USA: a population-based study. Lancet Digit Health.

[24] Sardá-Espinosa A (2019). Time-series clustering in R using the dtwclust package. The R Journal.

[25] Begum N, Ulanova L, Dau HA, Wang J, Keogh EJ (2016). A general framework for density based time series clustering exploiting a novel admissible pruning strategy. arXiv.

[26] Huh U, Tak YJ, Song S (2019). Feedback on physical activity through a wearable device connected to a mobile phone app in patients with metabolic syndrome: pilot study. JMIR Mhealth Uhealth.

[27] Sardá-Espinosa A (2023). Package 'dtwclust'. The Comprehensive R Archive Network.

[28] RStudio. Posit.

[29] Caspersen CJ, Pereira MA, Curran KM (2000). Changes in physical activity patterns in the United States, by sex and cross-sectional age. Med Sci Sports Exerc.

[30] To QG, Stanton R, Schoeppe S, Doering T, Vandelanotte C (2022). Differences in physical activity between weekdays and weekend days among U.S. children and adults: cross-sectional analysis of NHANES 2011-2014 data. Prev Med Rep.

[31] Malone SK, Patterson F, Grunin L (2021). Habitual physical activity patterns in a nationally representative sample of U.S. adults. Transl Behav Med.

[32] Rossen J, Hagströmer M, Larsson K, Johansson UB, von Rosen P (2022). Physical activity patterns among individuals with prediabetes or type 2 diabetes across two years-a longitudinal latent class analysis. Int J Environ Res Public Health.

[33] Montaruli A, Castelli L, Mulè A (2021). Biological rhythm and chronotype: new perspectives in health. Biomolecules.

[34] Henson J, Rowlands AV, Baldry E (2020). Physical behaviors and chronotype in people with type 2 diabetes. BMJ Open Diabetes Res Care.

[35] Lotti S, Pagliai G, Colombini B, Sofi F, Dinu M (2022). Chronotype differences in energy intake, cardiometabolic risk parameters, cancer, and depression: a systematic review with meta-analysis of observational studies. Adv Nutr.

[36] Zhang R, Cai X, Lin C (2022). The association between metabolic parameters and evening chronotype and social jetlag in non-shift workers: a meta-analysis. Front Endocrinol (Lausanne).

[37] Lee IM, Sesso HD, Oguma Y, Paffenbarger RS Jr (2004). The “weekend warrior” and risk of mortality. Am J Epidemiol.

[38] Jang YS, Joo HJ, Jung YH, Park EC, Jang SY (2022). Association of the “weekend warrior” and other physical activity patterns with metabolic syndrome in the South Korean population. Int J Environ Res Public Health.

[39] O’Donovan G, Sarmiento OL, Hamer M (2018). The rise of the "weekend warrior". J Orthop Sports Phys Ther.

[40] Xiao J, Chu M, Shen H (2018). Relationship of “weekend warrior” and regular physical activity patterns with metabolic syndrome and its associated diseases among Chinese rural adults. J Sports Sci.

