# Supplementary material for: Physical Activity Pattern of Adults With Metabolic Syndrome Risk Factors: Time-Series Cluster Analysis
Source: JMIR Mhealth Uhealth. 2023 Dec 1;11:e50663. doi: 10.2196/50663 (PMC10718482; doi:10.2196/50663)
Supplement: Multimedia Appendix 1 [file mhealth-v11-e50663-s001.docx]

**Appendix**

Table S1. Wearable models worn by study participants

| Wearable model |  | No. participants |
| --- | --- | --- |
| Apple Watch series |  | 15 |
|  | Apple Watch 3 | 1 |
|  | Apple Watch 4 | 2 |
|  | Apple Watch 5 | 1 |
|  | Apple Watch 6 | 4 |
|  | Apple Watch 7 | 1 |
|  | Apple Watch SE | 6 |
| Samsung Galaxy Watch series |  | 19 |
|  | Galaxy Fit 1 | 3 |
|  | Galaxy Fit 2 | 6 |
|  | Galaxy Watch 1 | 1 |
|  | Galaxy Watch 2 | 2 |
|  | Galaxy Watch 3 | 1 |
|  | Galaxy Watch 4 | 4 |
|  | Galaxy Watch Active | 1 |
|  | Galaxy Watch Active 2 | 1 |
| Xiaomi Mi Band series |  | 13 |
|  | Mi Band 3 | 6 |
|  | Mi Band 4 | 2 |
|  | Mi Band 5 | 1 |
|  | Mi Band 6 | 3 |
|  | Xiaomi Amazfit GTR | 1 |

Table S2. Evaluation and diagnostic results of regression model

| McFadden’s Pseudo R^2^ | Accuracy | Hosmer-Lemeshow test | AUC |
| --- | --- | --- | --- |
| .2235 | .7827 | *P*=.9592 | .8202 |


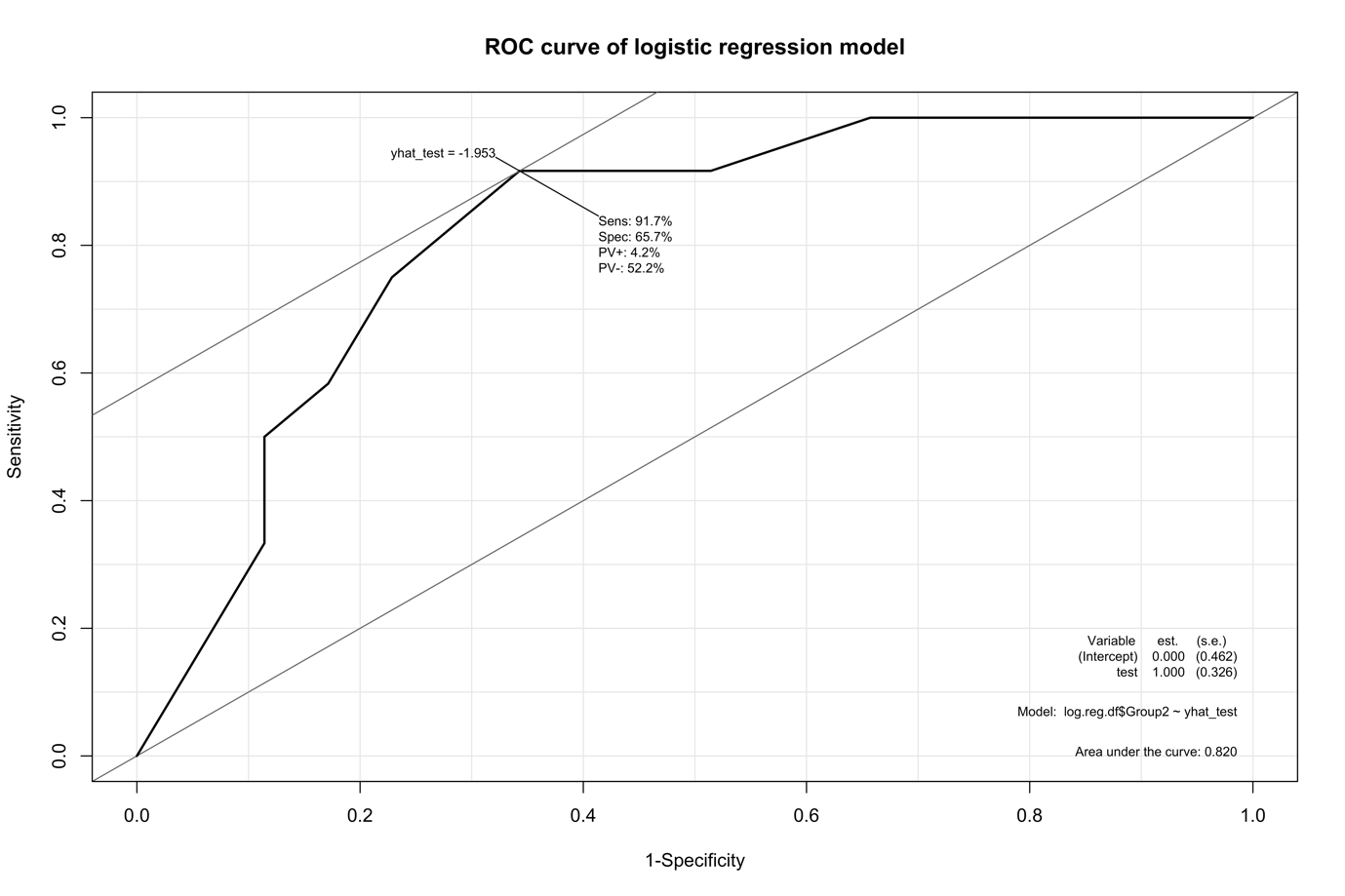


Figure S1. ROC curve

Table S3. Evaluation and diagnostic results of regression model for opposite direction

| McFadden’s Pseudo R^2^ | Accuracy | Hosmer-Lemeshow test | AUC |
| --- | --- | --- | --- |
| .0823 | .6383 | *P*=.9998 | .6818 |


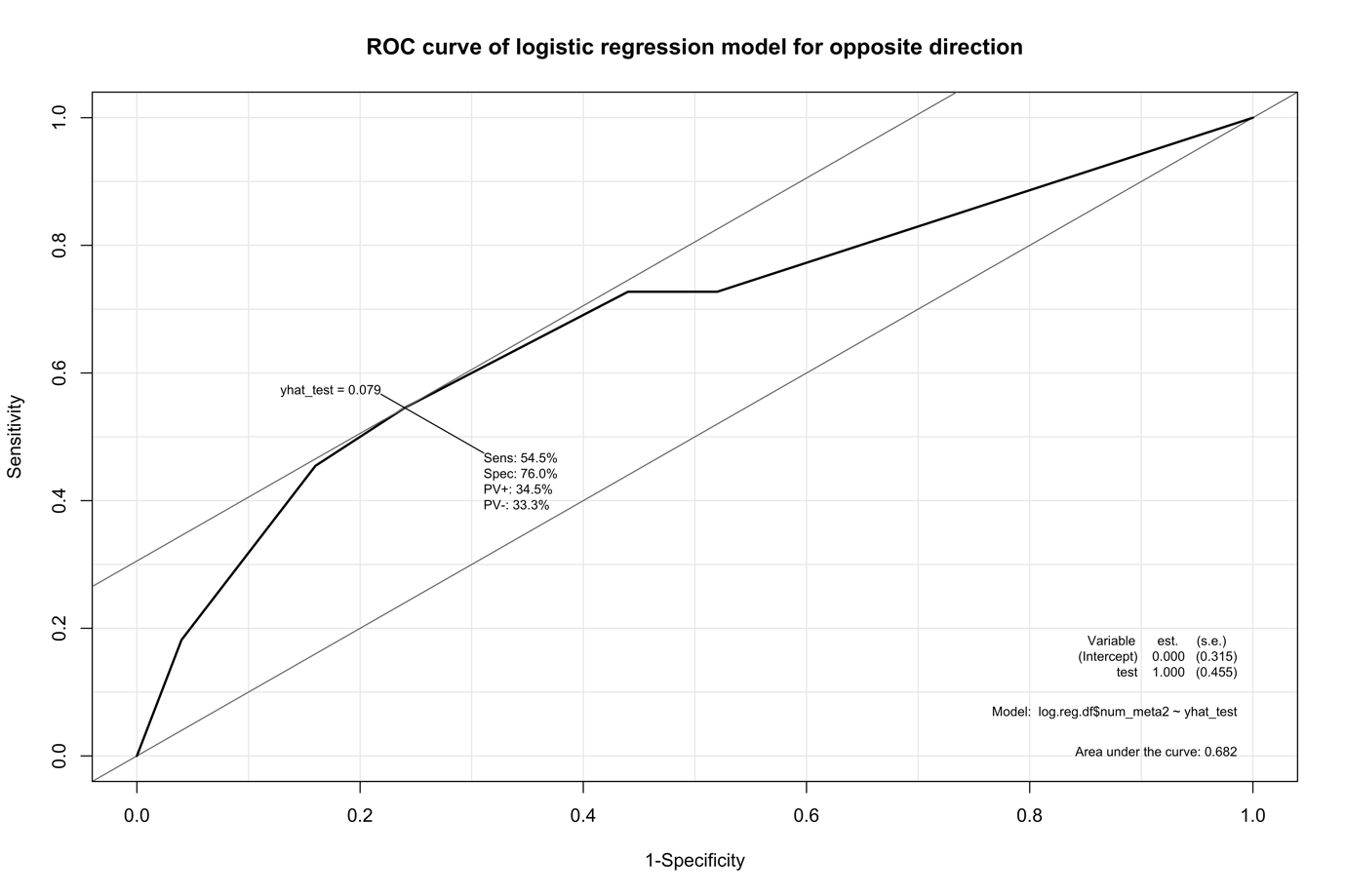


Figure S2. ROC curve of logistic regression model for opposite direction
